# Artificial Intelligence and Online Dermatology Misinformation May Mislead Both Patients and Clinicians

**DOI:** 10.1016/j.jdin.2026.04.001

**Published:** 2026-04-11

**Authors:** Isabella J. Tan, Tarek Zieneldien, Sophia Ma, Janice Kim, Jane M. Grant-Kels

**Affiliations:** a Rutgers Robert Wood Johnson Medical School, 125 Paterson St, New Brunswick, NJ 08901; b Johns Hopkins University School of Medicine, Baltimore, Maryland; c Michigan State University College of Osteopathic Medicine, East Lansing, Michigan; d Department of Dermatology, University of Connecticut, Farmington, Connecticut; e Department of Dermatology, University of Florida, Gainesville, Florida

**Keywords:** AI, artificial intelligence, digital literacy, ethical practice, ethics, evidence-based dermatology, Instagram, natural remedies, patient autonomy, patient education, Reddit, social media misinformation, TikTok, topical steroids, YouTube

**Figure:**
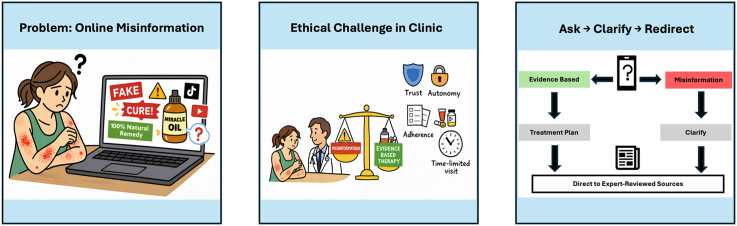

*To the Editor:* Over 60% of dermatology patients now search online, and many encounter authoritative-sounding but incorrect claims.^1^^,^^2^ Patients may then present with inaccurate convictions, request ineffective or harmful treatments, or decline physician-suggested evidence-based therapy. A recent national survey found that 86% of physicians believe medical misinformation has worsened over the last 5 years, impairing care.^3^ Emerging artificial intelligence (AI)–generated hallucinations further amplify misinformation into scientific literature, creating shared uncertainty for clinicians. Social media and AI-fabricated content erode patients’ factual foundation, undermining trust in physicians, especially in a service-oriented health care environment where patients increasingly evaluate care as a consumer experience. Factors contributing to the popularity of this resource to our patients include ease of access, availability, algorithmic amplification, and the perception that physician guidance may be optional, hard to apply to daily decisions, or less understandable than online sources. True patient autonomy depends on accurate and comprehensible information; persuasive falsehoods can override professional guidance.^4^ Patients’ trust is therefore threatened by misinformation itself, as well as by the perception that medical expertise is a service commodity rather than an authoritative and evidence-based resource.

The dermatologist’s professional duty in these scenarios should include correction of misinformation affecting patient decisions, identification of claims conflicting with clinical consensus or guidelines, redirection toward vetted, expert-reviewed resources, and acknowledgment that some published materials may be unreliable. A brief intake question, such as “Have you seen anything online about this condition or treatment?” can surface misconceptions early. Clinicians and patients should recognize that even reputable online resources may be influenced by AI-generated content or outdated information. Relying on curated, expert-reviewed sites, such as the *American Academy of Dermatology* patient pages, National Eczema Association, and National Psoriasis Foundation, provides the most reliable guidance while minimizing exposure to AI-amplified errors. Clinicians can also briefly review sources with patients to highlight which recommendations are accurate and evidence based.

Ethical care requires recognizing misinformation while maintaining trust. Recognizing misinformation involves identifying claims that are unsupported, inaccurate, or misleading, whereas learning new information entails assimilating validated and evidence-based knowledge. In practice, patients may bring both misinformation that requires correction and accurate, evidence-based updates they have encountered. Clinicians must therefore distinguish between correcting misconceptions, confirming accurate data, and guiding patients toward reliable new information. Understanding why patients sometimes rely on social media more than clinicians, including convenience, accessibility, and the perception that professional guidance may be less immediately applicable, can refine communication strategies and strengthen the clinician-patient relationship in an increasingly service-oriented context.

## Conflicts of interest

None disclosed.
